# The normal human chondro-osseous junctional region: evidence for contact of uncalcified cartilage with subchondral bone and marrow spaces

**DOI:** 10.1186/1471-2474-7-52

**Published:** 2006-06-20

**Authors:** Tim J Lyons, Sheena F McClure, Robert W Stoddart, John McClure

**Affiliations:** 1 Department of Forensic Medicine, Royal Newcastle Hospital, NSW, Australia; 2 Division of Laboratory and Regenerative Medicine, School of Medicine, The University of Manchester, UK; 3 Department of Musculoskeletal Pathology, RJAH Orthopaedic and District Hospital NHS Trust, Oswestry, Shropshire, SY10 7AG, UK

## Abstract

**Background:**

The chondro-osseous junctional region of diarthrodial joints is peculiarly complex and may be considered to consist of the deepest layer of non-calcified cartilage, the tidemark, the layer of calcified cartilage, a thin cement line (between the calcified cartilage and the subchondral bone) and the subchondral bone. A detailed knowledge of the structure, function and pathophysiology of the normal chondro-osseous junction is essential for an understanding of the pathogenesis of osteoarthrosis.

**Methods:**

Full thickness samples from human knee joints were processed and embedded in paraffin wax. One hundred serial sections (10 μm thick) were taken from the chondro-osseous junctional region of a block from the medial tibial plateau of a normal joint. They were stained with haematoxylin and eosin and photographed. For a simple physical reconstruction images of each 10th sequential tissue section were printed and the areas of the photomicrographs containing the chondro-osseous junctional region were cut out and then overlaid so as to create a three-dimensional (3D) model of this region. A 3D reconstruction was also made using computer modelling.

**Results:**

Histochemical staining revealed some instances where prolongations of uncalcified cartilage, delineated by the tidemark, dipped into the calcified cartilage and, in places, abutted onto subchondral bone and marrow spaces. Small areas of uncalcified cartilage containing chondrocytes (virtual islands) were seen, in two-dimensional (2D) sections, to be apparently entombed in calcified matrix. The simple physical 3D reconstruction confirmed that these prolongations of uncalcified cartilage were continuous with the cartilage of zone IV and demonstrated that the virtual islands of uncalcified cartilage were cross-sections of these prolongations. The computer-generated 3D reconstructions clearly demonstrated that the uncalcified prolongations ran through the calcified cartilage to touch bone and marrow spaces and confirmed that the apparent entombment of chondrocytes was a 2D artefact.

**Conclusion:**

This study demonstrates that the chondro-osseous junctional region is more complex than previously described. The tidemark is a clearly defined boundary delineating uncalcified from calcified cartilage. It is not a straight line across a joint, but a complex three-dimensional structure that follows uncalcified cartilage prolongations dipping down through the calcified cartilage to abut onto subjacent bone or marrow spaces.

## Background

**Figure 1:**
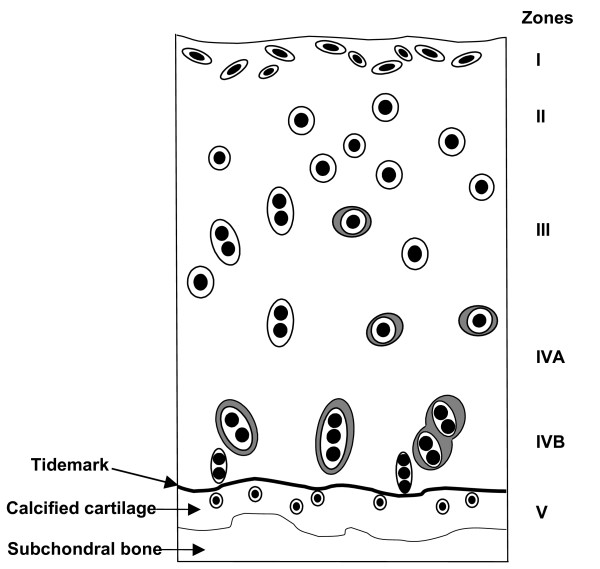
**Diagrammatic representation of the zones of articular cartilage and subchondral bone**. (Zones as per Gardner *et al*. 1987 [13])

The integrity of the junctions between different tissues is important in health and disease, but generally these structures are poorly understood. The chondro-osseous junctional region (COJ) of diarthrodial joints is peculiarly complex and may be considered to consist, in its full extent and in sequence, of the deepest layer of non-calcified cartilage (zone IVB), the tidemark, the layer of calcified cartilage (zone V), a thin line (which may be inapparent) between the calcified cartilage and the subchondral bone and which is sometimes referred to as a cement line, the subchondral plate of lamellar bone, trabecular bone and bone marrow spaces (Figure 1). A detailed knowledge of the structure, function and pathophysiology of the normal chondro-osseous junction is essential for an understanding of the pathogenesis of osteoarthrosis.

The tidemark is the junction of calcified and uncalcified cartilage matrix and it is traditionally described as a haematoxyphil single line up to 10 μm in thickness [1]. Since replication of the tidemark is taken to be a feature of the osteoarthrotic process [2], representing the advance of a calcification front into the uncalcified cartilage of zone IV, this structure is better known than other components of the COJ. There are limited data available on the layer of calcified cartilage.

Many of the previous investigations of the COJ have involved human material from advanced osteoarthrosis and animal models. As part of a larger study material was collected from the human knee joint at necropsy or during arthroplasty. All the cases had been assessed clinically, macroscopically and microscopically against a number of criteria to determine the presence and degree of osteoarthrosis, age-related changes or the complete absence of accepted disease features ('normality'). In the series there were a number of cases categorised as normal. Observations of histological sections of these normal knee joints showed that they contained previously unrecorded anatomical structures in the COJ. To study the significance of the observations we performed three-dimensional (3D) reconstructions of the COJ. The results of this stereological study are reported herein and their significance is discussed.

## Methods

Knee joints were removed from necropsy cases (with the informed consent of the next of kin) and material was obtained, again with informed consent, from knee joint arthroplasties. Specimens were sectioned on a band saw in an anteroposterior direction across the centre of each compartment, photographed and assessed macroscopically.

Full thickness samples (cartilage and bone) were taken from seven defined anatomical regions viz, femoral intercondylar notch, anterior lateral femoral condyle, posterior lateral femoral condyle, anterior medial femoral condyle, posterior medial femoral condyle, medial tibial plateau and lateral tibial plateau for each case. These were fixed in isotonic formaldehyde acetic acid (pH 2.8), decalcified in 0.19 M EDTA (pH 7.4–7.6 for 2–7 days with X-ray control) and embedded in paraffin wax by standard procedures. Sections (7 μm) were stained by haematoxylin and eosin (H&E), toluidine blue (alcoholic), safranin O, alcian blue in critical electrolyte concentrations of 0.05 M, 0.5 M and 0.9 M magnesium chloride [3] and picro-sirius red. All the cases were assessed clinically and then macroscopically and microscopically for the following features: surface – smooth; matrix – uniform, pale eosinophilic in zones I-III, more eosinophilic in zone IV and strongly eosinophilic in zone V; chondrocytes – rounded and in arcades in deep zones, elliptical/flat in upper zones, pale cytoplasm, eosinophilic membrane; tidemark present as a intact single line with no splits. The presence of these features indicates that the cartilage is normal and free of the characteristics of osteoarthrotic disease or ageing. Three of the cases had sections from all their blocks categorised as normal. One block from the medial tibial plateau of one of these cases (necropsy, male aged 27) was used in the 3D reconstruction. One hundred serial sections (10 μm thick) were taken from the COJ of this block.

They were stained with haematoxylin and eosin and images recorded via a Sony CCD video camera mounted on an Olympus BNZ microscope. Each image had 768 × 575 pixels with eight bits of grey-level information. For an initial simple physical reconstruction black and white images of each 10th sequential tissue section were recorded and printed. The areas of the photomicrographs containing the COJ were cut out along the edges of the tidemark and the cement line and following along the edges of any uncalcified cartilage prolongations. These photomicrographs were then overlaid so as to create a 3D model of the COJ.

For a computer-driven reconstruction the images of 50 serial sections were used. The digitised images were converted to a mathematical algorithm that allowed alignment to 3D voxels and the construction of a 3D image. Essentially, grey scales were converted to colour. The work was performed on a Unix-operated silicon graphics indigo workstation, using in-house software developed by the Department of Physics, University of Manchester, for volumodensimetric studies.

## Results

**Figure 2:**
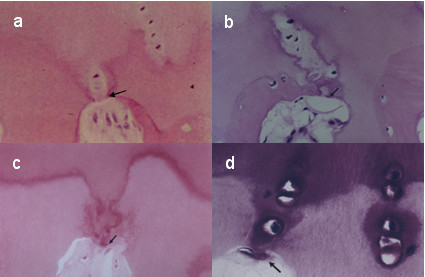
**Histochemical staining of the COJ region of normal cartilage**. (a & b) with H&E, (c) picro-sirius red and (d) toluidine blue. Prolongations of uncalcified cartilage are seen to dip down through the calcified cartilage and abut the marrow spaces at points indicated by the arrows.

**Figure 3:**
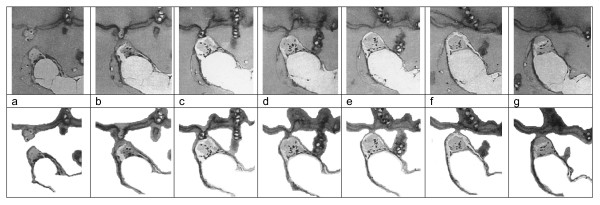
**3D reconstruction of the tidemark**. Sequential sections (a-g) from a normal COJ region demonstrate the continuity of apparently discontinuous non-calcified cartilage prolongations which protrude into the calcified cartilage and abut onto a bone marrow space. The lower set of photographs has been edited to show more clearly the sections through the prolongations. Sections were 10 μm thick and every 10^th ^section is shown.

**Figure 4:**
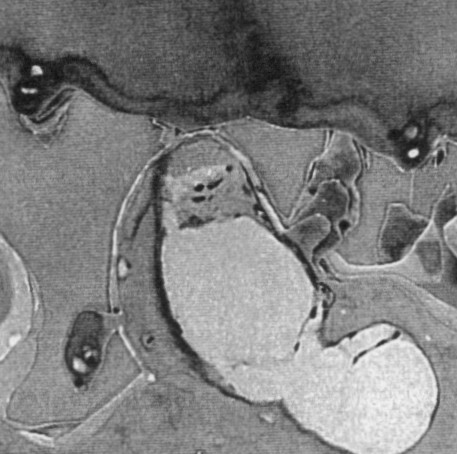
**3D reconstruction of the tidemark**. This shows a composite photograph made by cutting around the cartilage prolongations on the individual photographs from Figure 3 and then overlaying them.

**Figure 5:**
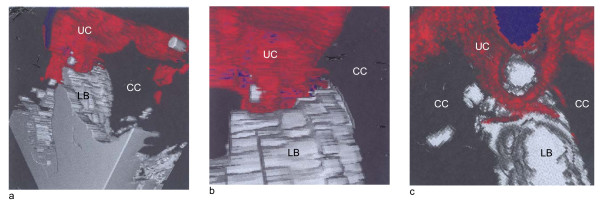
**Computer generated 3D reconstruction of the COJ**. Panoramic view (a), lateral view (b) and superior view (c). In (a) contacts between uncalcified cartilage (UC – red), calcified cartilage (CC – dark grey) and lamellar bone (LB – grey) are evident. In (b) and (c) contact between uncalcified cartilage and lamellar bone is illustrated.

**Figure 6:**
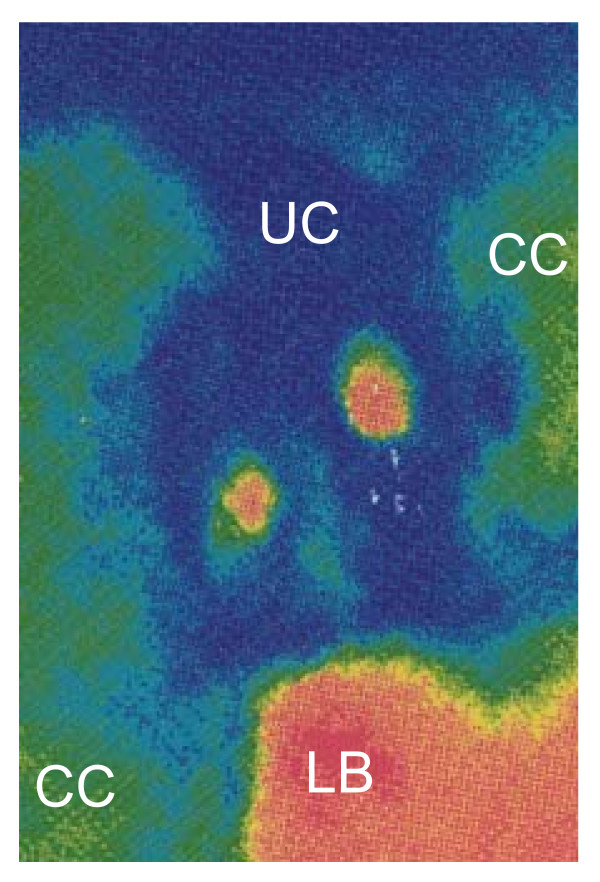
**Computer generated image of the COJ**. Uncalcified cartilage (UC) is dark blue, calcified cartilage (CC) is green and lamellar bone (LB) is red. The image represents the abutment of uncalcified cartilage onto lamellar bone.

Histochemical staining revealed instances where prolongations of uncalcified cartilage (from zone IVB), delineated by the tidemark, dipped into the calcified cartilage (zone V) and, in places, abutted onto subchondral bone and marrow spaces (Figure 2). Small areas of uncalcified cartilage containing chondrocytes were seen, in 2D sections, to be apparently entombed in the calcified cartilage of zone V. These were termed virtual islands. These prolongations and virtual islands were seen in all the normal cases and in all the sampled anatomical regions of the knees. The simple physical 3D reconstruction (Figures 3 and 4) showed an area of the COJ in serial sections and demonstrated the continuity of one of the prolongations through the tissue of zone V cartilage. It confirmed that the prolongation of uncalcified cartilage was continuous from the cartilage of zone IV to the subchondral bone. It also demonstrated that the virtual islands of uncalcified cartilage were cross-sections of these prolongations. The prolongations appeared to be slightly helical in shape. The computer generated 3D reconstructions clearly demonstrated that the uncalcified prolongations ran through the calcified cartilage to touch bone and marrow spaces and confirmed that the apparent entombment of small areas of uncalcified cartilage matrix, containing chondrocytes, was a 2D artefact (Figures 5 and 6). Chondrocytes within both the prolongations and the virtual islands were invariably morphologically viable.

## Discussion

**Figure 7:**
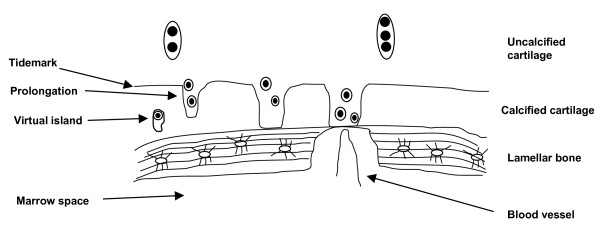
**Revised representation of the components of the chondro-osseous junctional region of articular cartilage**. This shows a virtual island, one prolongation of the uncalcified cartilage into the calcified cartilage layer, one prolongation that abuts the bone and one prolongation that abuts the marrow space containing a blood vessel.

Because of the relative paucity of studies clearly defining the tidemark and subjacent structures in normal cartilage, we were prompted to perform 3D reconstructions, both physical and computer-generated. A revised model of the anatomy of the COJ is proposed in the light of the observations of these reconstructions (Figure 7).

Previous research on the COJ has focused on the tinctorial properties of the tidemark [4-6], its ultrastructure [7] and its role as a mineralisation front [8,9]. Some authors have considered it as a membrane that runs across the joint and merges with the periosteum [2] and others as a zone of least resistance [10]. A study by Teshima *et al *[11], using 3D images, demonstrated irregularities of the surface of the subchondral bone plate at the COJ in femoral heads. They showed that these irregularities were greater in the higher weight-bearing regions, but they did not show the tidemark or any connection between the tidemark and the subchondral bone. Another stereological study of the tidemark in patellar cartilage measured the surface area of the tidemark [12], but again did not demonstrate the prolongations found in our study. Very few studies have focused on the overall micro-anatomy of the COJ and the significance that this might have for its normal physiological role. The present study prompts re-interpretation of the light-microscopical features, suggesting that the COJ is a more complex region than has been commonly accepted. This study clearly demonstrated that prolongations of uncalcified cartilage interdigitated with the calcified cartilage and that these were faithfully followed by the tidemark and abutted onto subchondral lamellar bone or marrow spaces.

The 3D reconstructions were initially carried out using a simple physical model in which a series of sequential photomicrographs were taken of a normal tidemark region, 'cut out' and overlaid. This straightforward technique clearly demonstrated the continuity of uncalcified cartilage prolongations through the calcified zone. There are no previous data from 3D reconstructions showing these prolongations in the calcified cartilage. The other reconstruction technique that we applied had been developed for the assessment of 'hot' and 'cold' spots in radiation dose fields in tumour therapy. In this system 50 serial black and white images fed from a microscope-mounted, computer-controlled digital camera allowed reconstruction of the region. Grey scales were converted to colour images and, using appropriate computer algorithms, the data were rendered into 3D images.

Again these reconstructions clearly demonstrated prolongations of uncalcified cartilage dipping through the calcified cartilage and impinging on lamellar bone or marrow spaces. Resolution was not sufficient to demonstrate the tidemark *per se *as a separate entity. However, histological studies indicated that the tidemark always followed these prolongations. The apparent islands of uncalcified cartilage seen in 2D sections are parts of these prolongations from the uncalcified cartilage and the viability of their constituent cells may be maintained by diffusion from the latter. The apparent helical shape of the prolongations would explain why these prolongations were not seen as complete structures in 2D sections. These structures join uncalcified hyaline cartilage to marrow spaces and could provide a molecular diffusion pathway (potentially from synovium/joint space to marrow and vice versa), which may have a nutritional and metabolic role. In addition, this area is profoundly affected in osteoarthrosis and humoral mediators may affect cartilage matrix components *via *these communications. Direct contact between uncalcified cartilage and lamellar bone also allows opportunities for molecular trafficking and/or the transmission of physical forces. The helical shape may be of some biomechanical significance in allowing for small compressions of calcified cartilage and in preventing fracture between calcified and uncalcified layers of cartilage.

From our studies it is clear that the chondro-osseous junctional region is more complex than previously described (Figure 7). The tidemark is a clearly defined boundary delineating uncalcified from calcified cartilage. It is not a straight line across a joint, but a complex 3D structure that follows uncalcified cartilage prolongations, dipping down through the calcified cartilage to abut onto subjacent bone or marrow spaces. Based on the current and previous studies, it is postulated that this region is involved in joint nutrition, mineralisation and the transfer of forces from hyaline cartilage to bone.

Replication of the tidemark is considered to represent an advance of the calcification front into zone IV cartilage. If this is the case then the calcification process must be determined and regulated by adjacent chondrocytes. In general chondrocytes near the tidemark must maintain control over the matrix domain in their proximity and regulate the turnover of non-collagenous components of this matrix. In the normal joint, the calcification front behaviour of the tidemark is necessarily quiescent, since prevention of all the articular cartilage from becoming calcified is an obvious prerequisite for joint function. The mechanisms involved have barely been investigated.

Irrespective of the site of initiating stimulus (articular cartilage or subchondral bone) advancing osteoarthrosis is characterised by an endochondral ossification process – advancing calcification of zone IV and replacement of calcified cartilage by new bone at the calcified cartilage-bone interface, and these events must be precisely regulated and co-ordinated. The model of the COJ proposed herein provides micro-anatomical pathways for the molecular trafficking necessary for this regulation and co-ordination.

## Conclusion

This study demonstrated that the chondro-osseous junctional region is more complex than previously described. The tidemark is a clearly defined boundary delineating uncalcified from calcified cartilage. It is not a straight line across a joint, but a complex 3D structure that follows the prolongations of uncalcified cartilage from zone IV, dipping down through the calcified cartilage to abut onto subjacent bone or marrow spaces. The islands of uncalcified cartilage seen in 2D tissue sections are virtual islands and the 3D reconstructions show these to be cross sections of the uncalcified cartilage prolongations.

## Abbreviations

COJ = chondro-osseous junctional region; 3D = three-dimensional; 2D = two-dimensional; H&E = haematoxylin and eosin.

## Competing interests

The author(s) declare that they have no competing interests.

## Authors' contributions

TJL obtained the tissue samples, performed the 3D reconstructions and participated in the interpretation of the results and the drafting of the manuscript. SMcC participated in the interpretation of the results and drafted the manuscript. RWS participated in the interpretation of the results and in the final drafting of the manuscript. JMcC conceived the study, participated in its design and helped in the drafting and final editing of the manuscript. All authors read and approved the final manuscript.

## Pre-publication history

The pre-publication history for this paper can be accessed here:
